# Glass Shape Influences Consumption Rate for Alcoholic Beverages

**DOI:** 10.1371/journal.pone.0043007

**Published:** 2012-08-17

**Authors:** Angela S. Attwood, Nicholas E. Scott-Samuel, George Stothart, Marcus R. Munafò

**Affiliations:** School of Experimental Psychology, University of Bristol, Bristol, United Kingdom; Federal University of Rio de Janeiro, Brazil

## Abstract

**Background:**

High levels of alcohol consumption and increases in heavy episodic drinking (binge drinking) are a growing public concern, due to their association with increased risk of personal and societal harm. Alcohol consumption has been shown to be sensitive to factors such as price and availability. The aim of this study was to explore the influence of glass shape on the rate of consumption of alcoholic and non-alcoholic beverages.

**Methods:**

This was an experimental design with beverage (lager, soft drink), glass (straight, curved) and quantity (6 fl oz, 12 fl oz) as between-subjects factors. Social male and female alcohol consumers (*n* = 159) attended two experimental sessions, and were randomised to drink either lager or a soft drink from either a curved or straight-sided glass, and complete a computerised task identifying perceived midpoint of the two glasses (order counterbalanced). Ethical approval was granted by the Faculty of Science Research Ethics Committee at the University of Bristol. The primary outcome measures were total drinking time of an alcoholic or non-alcoholic beverage, and perceptual judgement of the half-way point of a straight and curved glass.

**Results:**

Participants were 60% slower to consume an alcoholic beverage from a straight glass compared to a curved glass. This effect was only observed for a full glass and not a half-full glass, and was not observed for a non-alcoholic beverage. Participants also misjudged the half-way point of a curved glass to a greater degree than that of a straight glass, and there was a trend towards a positive association between the degree of error and total drinking time.

**Conclusions:**

Glass shape appears to influence the rate of drinking of alcoholic beverages. This may represent a modifiable target for public health interventions.

## Introduction

Alcohol consumption is associated with increased mortality and morbidity, with approximately 4% of the global burden of disease attributable to alcohol use [1]. In recent years, there has been particular concern regarding patterns of alcohol consumption in adolescents and young adults across several countries. Rates of heavy episodic drinking (binge drinking) have increased in this group in the United Kingdom and elsewhere, in particular in young women [2], [3], with corresponding increases in the risk of personal and societal harm. Furthermore, estimates of drinking behaviour that are based on standard units of alcohol may underestimate levels of consumption. Gill and Donaghy [4] report that drinkers were more likely to self-pour single drink servings that were in the region of two units of alcohol, indicating that a “drink” is not necessarily equivalent to a “unit”. Individuals may also underestimate the alcohol content of beverages as ostensibly similar classes of beverage (e.g., beer) vary widely in alcohol content, and therefore drinkers may be less able to gauge their consumption during a drinking episode and may underestimate the amount of alcohol that they have consumed [5]. There is a need to better understand the factors influencing alcohol consumption and binge drinking, in order to inform policy and enable the design of public health interventions.

Alcohol consumption is known to be sensitive to factors such as price, with price elasticities (i.e., the change in demand for a product in response to a change in price) in the United Kingdom estimated as −0.48 for beer consumed on premises, meaning that a price increase of 10% (e.g., through increased taxation) would result in a decrease in consumption of 4.8% [6], and therefore a corresponding decrease in alcohol-related harms. Similar price elasticities have been observed for other alcoholic beverages [6]. Other restrictions on availability, such as increased drinking age, reduced hours and days when alcohol may be purchased, and reduced number, density and type of alcohol outlet, have also been shown to reduce consumption levels [1]. Unfortunately, despite evidence for the effectiveness of these alcohol control measures and apparent public support for implementation targeted controls [7], most governments have been unwilling to adopt many or all of them [8].

There may be other potentially modifiable factors which may influence alcohol consumption and drinking rate. These might include marketing signals (i.e., branding), and vehicles for these signals such as the glasses from which beverages are consumed. Legislation to control or limit these signals may therefore influence drinking behaviour. A parallel can be drawn with the tobacco control literature, where plain packaging has been shown to increase visual attention towards health warnings compared with branded packaging in non-smokers and light smokers [9].

In particular, there has been an increase in branded drinking glasses in the United Kingdom in recent years, many of which include shape as a differentiating feature. These glasses include chalice glasses, curved beer flutes, tankard and novel curved beer glasses, and have been used by numerous alcohol brands including Stella Artois, Heineken, Guinness, Pilsner, Amstel, Smirnoff, Carlsberg, Carling and Jameson's whiskey. While alcohol advertising is still permitted in the United Kingdom, packaging and, by extension, drinking glasses provide another, currently unregulated, marketing channel.

As well as presenting overt branding information, these glasses may influence drinking rate by disguising the volume of beverage remaining. Anecdotally, this is particularly the case in curved glasses where the majority of the volume is contained in the upper portion of the glass. There is also empirical research to support an influence of object shape on volume perception [10]. For example, there appears to be a bias to use information in the vertical plane when making judgements about the volume of an object, such that taller objects are judged as having a larger volume [11], [12], [13]. Volume perception has also been investigated in relation to marketing and consumer behaviour. Perceptual characteristics such as the height of the container may be used as “visual heuristics” to make judgements about the volume of the container [14]. Furthermore, Raghubir and Krishna [14] suggest that in contrast, perceived consumption is inversely related to object height, suggesting that individuals will perceive that they have consumed less from a taller container compared to a shorter one. There is also evidence indicating that glass shape can influence pouring practices. Wansink and van Ittersum [15] asked participants to pour a standard shot of alcohol, and found that both students and bar tenders tended to over-pour into a short wide glass compared to a slender glass of the same size. These findings indicate that object shape can influence not only the perceived volume of an object, but also behaviour towards the object when interacting with it.

To date there has been no research investigating the effect of glass shape on drinking behaviour. We therefore explored whether glass shape modifies the time taken to consume alcoholic and non-alcoholic beverages. We hypothesised that glass shapes where it is more difficult to accurately judge when half the beverage has been consumed would be associated with faster rates of drinking, compared with glass shapes where it is easier to make an accurate judgement.

## Methods

### Ethics statement

Ethical approval was granted by the Faculty of Science Research Ethics Committee at the University of Bristol and the study was conducted in accordance with the principles of the Declaration of Helsinki. Written informed consent was obtained from all participants.

### Design and Overview

Participants were allocated to one of eight groups using a factorial design in which they were asked to drink either 6 fl oz or 12 fl oz of either an alcoholic (lager) or a non-alcoholic (carbonated soft drink) beverage, from either a straight or curved 12 fl oz glass. Group allocation was randomised with the constraint that groups contained equal numbers of male and female participants. The experimental design therefore comprised three between-subjects factors of beverage (lager, soft drink), glass (straight, curved) and quantity (6 fl oz, 12 fl oz).

Participants attended two experimental sessions approximately one week apart. At one session participants were presented with a beverage and asked to consume this at their own pace whilst watching a nature documentary. These sessions were videotaped for subsequent analysis of consumptive behaviour. When they had finished the beverage, participants were given a wordsearch task that was included to disguise the aim of the study. During the other experimental session, participants completed a computer task designed to measure perceptual judgement of the fullness of each glass. A filler paper and pen task was included in order to further disguise the true nature of the study. Session order was randomised.

### Participants

One hundred and sixty (50% male) social alcohol drinkers (who reported consuming between 10 and 50 Units/week for males, and between 5 and 35 Units/week for females. One unit = a half pint (284 ml) of ordinary strength (3.5–4% ABV) beer, a small glass (125 ml) of 8% ABV of wine or a single measure (25 ml) of a 40% ABV spirit) were recruited from the staff and students of the University of Bristol and from the general population by means of poster and flyer advertisements, and word-of-mouth. Participants were required to be in good psychological and physical health, aged between 18 and 40 years, and not currently taking any psychiatric medication. Exclusion criteria included current use of illicit substances (excluding cannabis) and a direct family history of alcoholism (defined as parent and/or sibling) and not liking lager or lemonade. Eligibility was ascertained on these variables by self-report, i.e., participants were required to provide “Yes” or “No” responses to questions regarding their physical/psychiatric health, family history of alcoholism and current drinking quantities and preferences. Participants were asked to abstain from alcohol consumption for 12 hours prior to each test session, and were only enrolled onto the study if they provided a zero breath alcohol reading at the start of the session. Participants were paid £10 or awarded course credit, as appropriate, at the end of the study.

### Materials

#### Glasses and stimuli

**Figure 1:**
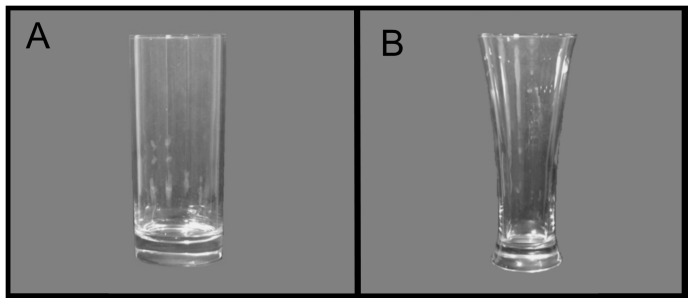
Straight-sided (A) and Curved (B) Glasses.

The two glasses were of equal volume (12 fl oz) and were both of a type frequently used to consume soft drinks and beer. One glass was straight-sided with an unambiguous midpoint (i.e., the halfway point in terms of height and volume are the same). The other glass was a curved flute-style glass with an ambiguous midpoint (i.e., the midpoint in terms of height and volume are not the same) (see Figure 1). This glass was chosen on the basis of a pilot study which demonstrated that its shape led to systematic misjudgement of its midpoint in terms of volume (data available on request). Both glasses were clear and did not include any markings.

For the computerised glass rating task, two sets of photographic stimuli were created using a digital camera (Canon Digital IXUS 70). Each set was a sequence of 61 photographs ranging from an empty (0) to a full (60) glass with liquid added in 60 equal volume increments. To do this, the total volume of the glass was measured and divided by 60. The photographs were converted to greyscale images (1024×768 pixels). Two interleaved 1-up 1-down staircases [16] were used to determine which stimulus to present on each trial. Participants were asked to judge whether the stimulus was more or less than half full, and responded by pressing designated keys on a keypad. The staircase method is an adaptive psychophysical procedure in which each stimulus presentation is dependent on previous responses. In the task used here, if a participant responded that the glass was less than half full, the next stimulus would show the glass with a greater volume of liquid; if the participant had responded that the glass was more than half full, the next image would show a glass with less liquid, and so on..The staircases converged on the level of liquid which they perceived as exactly half full. Stimuli were presented on a linearised Trinitron Ultrascan P991 monitor (Sony, Japan), driven by a Macintosh G5 computer, at a viewing distance of 128 cm. The experimental software was written in Matlab (MathWorks, Natick MA), using the Psychophysics Toolbox extensions [17], [18].

#### Video recording and analysis

The drinking session was recorded using a video camera (Hitachi Hybrid Camcorder DZHS500E). An independent researcher blind to the study hypotheses scored the video recordings of drinking behaviour, using a specifically-designed DOS program that involved pressing “1” each time a sip was initiated (defined as liquid touching lips) and “2” when the sip ended (defined as liquid leaving lips) [19]. To assess video analysis reliability of the primary outcome measure (total drinking time), 20% of video files were chosen at random and analysed by a second independent rater, who was blind to the study hypotheses. We conducted a reliability analysis, which is reported in the “results” section. Measures extracted included, total drink time (i.e., time from initiation of first sip to end of last sip), total sip duration and total sip interval (i.e., time between sips) duration, and total number of sips taken.

#### Filler task

To disguise the aim of the study, a filler task was included, where participants were asked to rate alcoholic and non-alcoholic beverages and branded bottles for likeability and alcohol-relatedness. The nature of the study was further disguised by inclusion of a 4-minute pen and paper wordsearch task, following consumption of the beverage. These data were not analysed.

#### Questionnaire measures

The questionnaire measures comprised the Alcohol Use Disorders Identification Test (AUDIT) [20], and the Alcohol Urges Questionnaire (AUQ) [21]. The AUDIT is a 10-item screening tool that was developed by the World Health Organization to identify hazardous and harmful drinking behaviour. Scores of 0–7 are generally considered low-risk, while scores of 8–14 are considered hazardous and scores of 15 or over are considered harmful. The AUQ is an 8-item state measure that assesses current urges to consume an alcoholic beverage. The AUQ scores were collected in order to use as covariates in the topography analysis, in order to ascertain whether any effects were driven by differences in urges to consume alcohol,

### Procedure

Experimental sessions lasted approximately 20 minutes and all testing took place between 12:00 and 18:00 hours. Upon arrival at their first session, participants were given an opportunity to read the information sheet and ask questions before providing written informed consent. Participants were told that the study examined the effects of alcohol consumption on wordsearch performance, in order to disguise the nature of the primary outcome measure of rate and pattern of drinking, which may have affected natural drinking behaviour. Participants were screened to ensure that they met the inclusion criteria, and provided a breath sample to ensure that they had complied with the alcohol abstinence requirement (i.e., zero breath alcohol reading).

For the drinking session, participants were randomised to receive either 6 fl oz (i.e., half a glass) or 12 fl oz (i.e., a full glass) of lager at 4% alcohol by volume (Bière de France, Brasserie de Saint-Omer) or soft drink (7-Up, Dr Pepper Snapple Group). All beverages were chilled prior to serving and were opened and poured immediately prior to consumption in order to ensure that carbonation was consistent across participants. Baseline ratings included self-report measures of alcohol use and craving (AUDIT, AUQ). Participants were then given their beverage and were told that they should consume all of it at their own pace whilst watching a nature documentary (Earth: The Journey of a Lifetime, BBC Worldwide 2007). A sequence of the footage was pre-selected that was considered not to contain emotive material. The experimenter started the videotape and left the room. When participants had finished their beverage, the experimenter returned and presented participants with a wordsearch task in which they were instructed to find as many words as possible in four minutes. Upon completion of this task, measures of alcohol craving (AUQ) were taken again.

For the computer session, the order of the computer tasks (i.e., curved glass, straight glass) was randomised. The straight and curved glass conditions of the computer tasks were presented in separate blocks. Between these blocks the picture-rating filler task was completed. At the end of the final session participants were informed that debriefing information would follow via email at the end of the study.

### Statistical Analysis

Data were analysed using a 2×2×2 ANOVA with beverage (lager, soft drink), glass (straight, curved) and quantity (6 fl oz, 12 fl oz) as between-subjects factors. For the analysis of AUQ data an additional within-subjects factor of time (pre-drinking, post-drinking) was included. Where significant interactions were observed, simple effects ANOVAs or t-tests (two tailed) were used as appropriate. Finally, to examine the association between drinking rate and perceptual bias, a Pearson's correlation analysis was conducted on the degree of perceptual bias of curved glass (i.e., difference from midpoint image) and the primary outcome measure of total drinking time. An alpha level of 0.05 was retained throughout.

## Results

### Characteristics of Participants

Data from one participant who was unable to finish the beverage were excluded. Participants (*n* = 159; 80 male) were on average aged 23 years (*SD* 4, range 18–37), weighed 71 kg (*SD* 12, range 46–110), and had an AUDIT score of 11 (*SD* 5, range 2–26). A series of 2×2×2 ANOVAs with beverage (lager, soft drink), glass (straight, curved) and quantity (6 fl oz, 12 fl oz) as between-subjects factors indicated no significant differences between groups on these measures (*p*s>0.13). Characteristics of participants are summarised in Table 1.

**Table 1:** Characteristics of Participants.

	Half Full Glass (6 fl oz)				Full Glass (12 fl oz)			
	Alcohol		Soft Drink		Alcohol		Soft Drink	
	Straight	Curved	Straight	Curved	Straight	Curved	Straight	Curved
	*n* = 20	*n* = 20	*n* = 20	*n* = 20	*n* = 20	*n* = 19	*n* = 20	*n* = 20
Age	24 (5)	23 (5)	24 (4)	24 (5)	22 (4)	22 (3)	24 (5)	22 (3)
Weight	68 (11)	72 (13)	72 (13)	68 (11)	72 (10)	72 (11)	71 (12)	74 (16)
AUDIT	11 (4)	11 (5)	10 (5)	11 (5)	12 (4)	11 (5)	11 (5)	11 (3)

Values represent mean (standard deviation).

### Perceptual Judgement Task

One-sample *t*-tests against a test value of 30 (reflecting the true half-way point on the perceptual judgement task) indicated that for both straight (*M* = 28, *SD* = 2, *t* [159] = 16.91, *p*<0.001) and curved (*M* = 21, *SD* = 3, *t* [159] = 37.31, *p*<0.001) glasses the half-way point was perceived to be below the true half-way point. A paired-sample *t*-test indicated a significant difference between the two glass conditions (*t* [159] = 30.89, *p*<0.001), indicating that the halfway-point was perceived to be below the true half-way point to a greater degree for the curved glass compared to the straight glass.

### Subjective Craving

A 2×2×2×2 ANOVA of AUQ data, with time (pre-drinking, post-drinking) as a within-subjects factor and beverage (lager, soft drink), glass (straight, curved) and quantity (6 fl oz, 12 fl oz) as between-subjects factors, indicated a main effect of time (*F* [1, 151] = 4.80, *p* = 0.030, partial η^2^ = .03), reflecting an increase in craving over time, and beverage (*F* [1, 151] = 5.86, *p* = 0.017, partial η^2^ = .04), reflecting greater craving in the lager group. These were qualified by a time × beverage interaction (*F* [1, 151] = 11.79, *p* = 0.001, partial η^2^ = .07), indicating that this increase in craving over time occurred in the lager condition but not in the soft drink condition. There was also a main effect of quantity (*F* [1, 151] = 5.14, *p* = 0.025, partial η^2^ = .03), reflecting higher craving in the 6 fl oz condition, but this was not qualified by interactions with time or beverage and therefore not considered further. There was no evidence for other main or interaction effects (*p*s>0.23).

### Drinking Topography

A 2×2×2 ANOVA of total drink time, with beverage (lager, soft drink), glass (straight, curved) and quantity (6 fl oz, 12 fl oz) as between-subjects factors, indicated main effects of beverage (*F* [1, 151] = 8.48, *p* = 0.004, partial η^2^ = .05), reflecting longer drinking time in the lager condition, glass (*F* [1, 151] = 4.93, *p* = 0.028, partial η^2^ = .03), reflecting longer drinking time in the straight condition, and quantity (*F* [1, 151] = 14.76, *p*<0.001. partial η^2^ = .09), reflecting shorter drinking time in the 6 fl oz condition. These were qualified by a beverage × glass × quantity interaction (*F* [1, 151] = 6.63, *p* = 0.011, partial η^2^ = .04).

**Figure 2:**
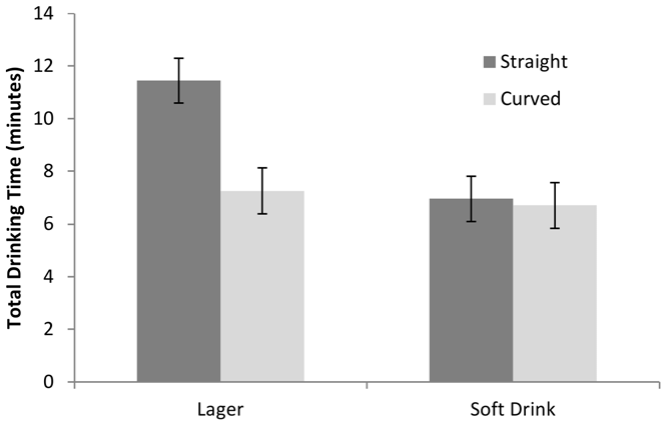
Total Drinking Time for Full (12 fl oz) Volume by Drink and Glass Type. Total drink time (minutes) for a 12 fl oz alcoholic or 12 fl oz non-alcoholic beverage is shown, grouped by glass type. Participants consuming an alcoholic beverage were slower to consume this when it was presented in a straight glass compared to a curved glass. This was not observed for participants consuming a non-alcoholic beverage. Error bars represent SEM.

To explore the beverage × glass × quantity interaction further, analyses were stratified by quantity. These indicated a beverage × glass interaction effect in the 12 fl oz condition (*F* [1, 75] = 5.30, *p* = 0.024, partial η^2^ = .07), but not the 6 fl oz condition (*F* [1, 76] = 1.84, *p* = 0.18, partial η^2^ = .02). Further stratified analyses indicated that total drinking time for 12 oz of lager was longer when consumed from a straight glass compared to a curved glass (*t* (37) = 2.83, *p* = 0.007, *d* = 0.93), but did not differ by glass condition for 12 oz of soft drink (*t* (38) = 0.29, *p* = 0.78, *d* = 0.09). Including change in subjective craving as a covariate did not alter these results. These data are presented in Figure 2.

To investigate the nature of this effect on drinking behaviour, additional 2×2×2 ANOVAs of total number of sips, total sip duration, total interval duration were conducted. There were main effects of glass for total number of sips (*F* [1, 151] = 5.97, *p* = 0.016, partial η^2^ = .04) and total interval duration (*F* [1, 151] = 4.75, *p* = 0.031, partial η^2^ = .03) reflecting more sips and longer time between sips in the straight versus curved glass condition. There were main effects of quantity for total number of sips (*F* [1, 151] = 52.21, *p*<0.001, partial η^2^ = .26), total sip duration (*F* [1, 151] = 61.00, *p*<0.001, partial η^2^ = .29) and total interval duration (*F* [1, 151] = 12.55, *p* = 0.001, partial η^2^ = .08) reflecting more sips and longer total sip and interval durations in the full versus half full condition. There was also a main effect of beverage for total interval duration (*F* [1, 151] = 8.54, *p* = 0.004, partial η^2^ = .05) with longer interval durations in the alcohol vs. placebo condition. There was a beverage × glass × quantity interaction for total interval duration (*F* [1, 151] = 6.87, *p* = 0.010, partial η^2^ = .04), which was explored further in analyses stratified by quantity. These indicated a beverage × glass interaction effect in the 12 fl oz condition (*F* [1, 75] = 5.41, *p* = 0.023, partial η^2^ = .07), but not the 6 fl oz condition (*F* [1, 76] = 1.97, *p* = 0.17, partial η^2^ = .03). Further stratified analyses indicated that total interval time for 12 fl oz of lager was longer when consumed from a straight glass compared to a curved glass (*t* = (37) 2.81, *p* = 0.008, *d* = 0.92), but did not differ by glass condition for 12 fl oz of soft drink (*t* (38) = 0.22, *p* = 0.83, *d* = 0.07).

### Correlational analysis

Three participants with outlier bias scores (ascertained using boxplots) were excluded from the analysis. There was a trend towards a significant positive correlation between total drinking time and perceptual bias to the curved glass, Pearson's *r*(156) = .15, *p* = .059.

### Reliability analysis

The ratings of the two independent raters were strongly and positively correlated, single measures intraclass correlation (31) = .98, *p*<.001, indicating a high level of inter-rater reliability.

## Discussion

Previous research has demonstrated differences in pouring practices depending on the glass shape. However, our study is the first to examine whether glass shape directly influences drinking behavior. Our data indicate that the shape of a drinking glass influences the rate of drinking of an alcoholic beverage, but not a non-alcoholic beverage. Specifically, alcoholic beverages were consumed more slowly from a straight glass than a curved glass when a full glass was presented. This effect was not observed when a half glass was presented, and was not observed for soft drinks. Moreover, participants under-estimated the true half-way point of a curved glass to a greater degree than a straight glass, and the degree of this bias was modestly associated with drinking rate (non-significant trend). This supports our initial hypothesis that glass shapes which induce a perceptual bias in the judgement of the half-way point in the glass will be associated with more rapid drinking. Additional analyses of topographical variables suggested that the slower drinking rate observed when consuming alcohol from a full, straight glass was due to more time spent between sips rather than due to differences in the number of sips or length of sips. These effects do not appear to be driven by differences in urges to drink alcohol, as the effect did not differ when change in AUQ scores were included as a covariate.

One possible mechanism for the effects we observed is that individuals may titrate their drinking rate based in part on perceptual judgements. That is, if they wish to consume a beverage in a certain time, they may titrate their drinking to reach the half-way point in the glass after half this time has elapsed. However, if their perceptual judgement of the half-way point is lower than the real value, they will have in fact consumed more than half of the beverage by this point (i.e., their drinking rate will be faster). Perceived consumption will affect subsequent drinking behaviour, as individuals who perceive that they have drunk little will compensate with an increase in consumption volume and/or rate [14]. Once a drinking rate has been established for a beverage, the total drinking time will be correspondingly reduced. Since the perceptual bias for curved glasses is a consequence of the top half of the glass being curved, while the bottom half is relatively straight, this explain why we only observed these effects for full glasses and not half-full glasses. An implication for policy is that drinking rate may be reduced if alcoholic beverages are served in glasses that have a clearly labeled midpoint, which enables the individual to more accurately gauge their drinking rate.

This does not explain why we observed this effect for alcoholic beverages but not non-alcoholic beverages. It may simply be that perceptual judgements play a greater role in influencing drinking rate for alcoholic beverages than for non-alcoholic beverages. It is known that dietary preferences are learned over time and become established, such that visual cues come to dominate food choice and portion size selection [22]. Due to expectancies of intoxication when consuming alcohol, individuals may be more motivated to regulate drinking rate of alcoholic beverages. As a result, they are more likely to use perceptual cues to inform behavior and “pace themselves” when drinking alcoholic beverages. Therefore, visual cues may differentially influence behaviour depending on whether they are associated with alcoholic or non-alcoholic beverages. It would be possible to test this directly by comparing beverages differing in alcohol content but matched for visual characteristics (e.g., alcoholic and non-alcoholic lager).

An alternative explanation is that the curved glass may have elicited alcohol-related conditioned responses and/or expectancies that influenced drinking behaviour. The curved glass is particularly associated with the consumption of lager beverages and this may have provided an additional cue that motivated alcohol consumption. The appetitive motivational model suggests that drug-related cues can elicit appetitive responses and positive affect, which in turn increase approach and consumptive behaviours. This is somewhat undermined by the fact the straight-sided glass is also commonly used to serve alcohol (i.e., it is similar to the standard half-pint glass used in many public houses). It can be argued however that the straight glass may be associated with the consumption of many different types of drink, including non-alcoholic drinks, and therefore the explicit association between the straight-sided glass and alcohol is weaker. This premise is merely speculative and further research is required to clarify the mechanism underlying the findings. These results have potential public health implications. A number of factors which might influence this have been considered, such as opening hours and the availability of low-cost alcohol [23]. It has been suggested that it is easier and more effective to influence licensed occupational behaviour than the behaviour of individual customers [1]. However, the role of more subtle factors such as glass shape has received little consideration. Our data indicate that total drinking time is slowed by almost 60% when an alcoholic beverage is presented in a straight glass compared with a curved glass. Clearly many other factors will influence drinking rate, including social context and the presence of others. However, even a modest reduction in drinking rate, when achieved across a large number of individuals, might lead to a substantial reduction in alcohol-related harm. There are some limitations to the present study which should be considered when interpreting these results. First, participants consumed their beverage alone, rather than in a social context. It is therefore unclear to what extent these findings would translate to a naturalistic environment where social drinking in groups is more usual. However, one study which investigated drinking topography as a function of social versus individual context found no differences in drinking rates in the two conditions [24]. In contrast, alcohol-related contexts have been shown to influence other relevant responses to alcohol and alcohol related cues, including decreased inhibitory control in heavy social drinkers [25] and decreased startle reflex magnitude in response to social drinking scenes in abstinent alcohol-dependent individuals [26]. While these aforementioned studies are informative, it should be noted that the manipulation of context was related to scenes presented in pictorial images, rather than changes to the participant's direct physical environment. Future studies should investigate the effects of glass shape on drinking rate in group conditions and in naturalistic settings, and should also consider the effects across a drinking session comprising ingestion of multiple drinks. Second, we only compared lager with a soft drink matched for carbonation. It would be desirable to explore similar effects across a range of alcoholic beverages. It would also be of interest to compare, for example, alcoholic and non-alcoholic lager, to determine whether the differential effects observed for alcoholic and non-alcoholic beverages in our study are due to visual characteristics of the beverages, or the presence or absence of the pharmacological effects of alcohol. Third, we used beverage volumes that are somewhat smaller than those typically served in the United Kingdom. This was in order to use glasses which differed in shape but not total volume. However, while it would be useful to replicate these findings with larger volumes, there are no particular reasons to think that our results would only apply to these smaller volumes. Finally, the mean AUDIT score of the participants was suggestive of hazardous drinking. The participants were all weekly consumers of alcohol and comprised many undergraduate students. Scores falling within the hazardous range are common in this population and these scores are comparable with findings from our previous research using this measure in these individuals. Future studies should replicate these findings in less frequent drinkers to examine the generalisability of the effect.

In conclusion, our data indicate that glass shape influences the rate of consumption of alcoholic beverages, possibly due to differences in the perceptual judgement of the half-way point of the glass across different glass shapes, although further research is required to examine the mechanisms underlying this effect. This, in turn, may influence pre-consumption decisions regarding the appropriate drinking rate necessary to titrate consumption as desired. While our study cannot fully resolve the mechanism which underlies the effects we observed, these findings have the potential to inform policy decisions regarding structural changes to the drinking environment which may reduce drinking rates and correspondingly impact on resulting alcohol-related harms.
